# Genome-Wide Identification of the *WUSCHEL-Related Homeobox* (*WOX*) Gene Family in Barley Reveals the Potential Role of *HvWOX8* in Salt Tolerance

**DOI:** 10.3390/ijms26052019

**Published:** 2025-02-26

**Authors:** Wenqi Zhang, Linli Huang, Longhua Zhou, Yingjie Zong, Runhong Gao, Yingbo Li, Chenghong Liu

**Affiliations:** 1 Biotechnology Research Institute, Shanghai Academy of Agricultural Sciences, 2901 Beidi Street, Shanghai 201106, China; wqzhang@saas.sh.cn (W.Z.); huanglinli@saas.sh.cn (L.H.); zhoulonghua@saas.sh.cn (L.Z.); 20170903@saas.sh.cn (Y.Z.); gaorunhong@saas.sh.cn (R.G.); liyingbo@saas.sh.cn (Y.L.); 2 Shanghai Key Laboratory of Agricultural Genetics and Breeding, Shanghai 201106, China

**Keywords:** *Hordeum vulgare*, *WUSCHEL-related homeobox*, plant regeneration, expression pattern, salt tolerance

## Abstract

The *WUSCHEL-related homeobox* (*WOX*) belongs to a plant-specific transcription factor gene family that plays crucial roles in plant growth and development. Barley ranks as the fourth global cereal crop and is recognized as a model crop for the study of cereal genetics. However, genome-wide characterization, functional validation, and stress-related studies of the *WOX* gene family in barley remain limited, hindering efforts to leverage their potential for improving salt tolerance and regeneration efficiency in breeding programs. In this study, we identified 12 *HvWOX* genes assigned from chromosome 1 to chromosome 5. Phylogenetic analysis revealed that these *HvWOX* genes can be classified into three clades (WUS, ancient, and intermediate). Gene structure analysis revealed that the exon numbers of *HvWOX* genes varied in the WUS and intermediate clades but were highly conserved in the ancient clade. Tissue-specific analysis revealed that the most common *HvWOX* genes were highly expressed in reproductive tissues such as anthers or ovaries. *Cis*-element analysis suggested that there were multiple stress- and hormone-responsive elements in the *HvWOX* gene promoters. In addition, overexpression of *HvWOX8* in *Arabidopsis* significantly enhanced root elongation under salt stress (50–100 mM NaCl), suggesting its direct role in salt tolerance. Transcriptomic analysis further revealed that *HvWOX8* modulates hormone signaling and electron transfer pathways during ATP synthesis under stress conditions. In conclusion, our results provided a comprehensive understanding of the gene characteristics, expression patterns, and potential roles of barley *WOX* genes.

## 1. Introduction

Plants have evolved remarkable abilities to regenerate whole plants from selected or genetically altered cells or tissues. Understanding the mechanisms behind this process can greatly increase fundamental research and expand the possibilities of plant biotechnology [1]. To this end, numerous studies have explored genes that impact in vitro plant regeneration, with some identifying potential candidates that affect somatic embryogenesis (SE). For example, *LEAFY COTYLEDONs* (*LECs*), *BABY BOOM* (*BBM*), and *SOMATIC EMBRYOGENESIS RECEPTOR KINASE* (*SERK*) have been identified and studied [2,3,4]. Among them, *WUSCHEL-related homeobox* (*WOX*) transcription factors are essential in various developmental processes in plants. They are involved in embryo patterning, embryonic polarization, stem-cell maintenance, lateral organ formation, seed formation, and regeneration of isolated tissues and organs [5,6]. The *WOX* gene family is a part of the plant superfamily of homeobox (HB) transcription factor families and is characterized by a short stretch of amino acids (60–66 residues) [7]. On the basis of the phylogenetic relationships among WOX proteins in plants, they can be classified into three clades: WUS, ancient clades, and intermediate clades [6,7].

The *WOX* gene family has been extensively studied across diverse species, including Arabidopsis, rice, wheat, barley, maize, soybean, poplar, and apple [5,6,8,9,10,11,12]. Functional studies reveal conserved and diversified roles:

Developmental regulation: *AtWUS* specifies shoot/floral meristem identity [13], while *AtWOX2/8* and *PaWOX2* regulate embryogenesis [14,15]. *OsWUS* drives tiller bud formation [16,17], *TaWOX5* enhances transformation efficiency in cereals [18], and *MdWOX4b* enhances adventitious root formation [12].

Stress and hormone responses: *PagWOX11*/*12a* improves salt tolerance via ROS scavenging [19], and *OsWOX11* integrates auxin/cytokinin signaling for crown root initiation [20]. Also, cytokinins regulate de novo shoot organogenesis by modulating gene expression, including key genes such as *WUS*, *CLV3*, and *ARR*, which are essential for shoot meristem formation and maintenance [21].

Stem cell maintenance: *AtWOX4* activates cambium activity [22], *AtWOX5* maintains root stem cells [23,24], and *AtWOX11/12* direct root organogenesis [25,26].

Despite functional overlaps, species- and member-specific differences exist [11,27], underscoring the need for systematic characterization in target species.

Given the conserved roles of *WOX* genes in plant development, regeneration, and stress adaptation, elucidating their functions in barley could provide critical insights for enhancing its agronomic traits and resilience under environmental challenges. Barley is the fourth largest cereal crop in the world and is used mainly for animal feed and malting. Barley has attracted considerable attention as a fiber-rich health food [28]. In recent years, the application of modern biotechnology tools in plant breeding has improved the yield and quality of major crops and enhanced their biological or abiotic resistance, but the realization of these goals has been limited by genetic transformation. To date, high frequencies of embryo formation and plant regeneration in isolated microspore cultures have been reported in barley [29]. An in-depth study of regeneration-related genes at the molecular level combined with microspore culture will help overcome the bottleneck in genetic transformation, shorten the number of breeding years and improve regeneration efficiency. Despite the identification of *WOX* genes in barley, previous studies in barley have primarily focused on *WOX* gene identification, yet comprehensive analyses of their expression dynamics, hormonal regulation, and functional roles in abiotic stress responses are lacking. Here, we characterized 12 putative *HvWOX* genes via a genome scanning approach. To investigate the potential functions of *HvWOX* genes, a genome-wide analysis was performed to study their physicochemical characteristics, gene structure, phylogenetic relationships, and expression patterns. We also analyzed the *cis*-regulatory elements and gene expression in different tissues under IAA and ABA conditions. Considering the expression pattern of *HvWOX8* in roots, we investigated the effect of *HvWOX8* on plant salt tolerance. This study paves the way for functional characterization of *HvWOX* genes and provides insights into their potential role in growth and development in barley.

## 2. Results

### 2.1. Identification and Chromosomal Distribution of HvWOX Genes

**Figure 1:**
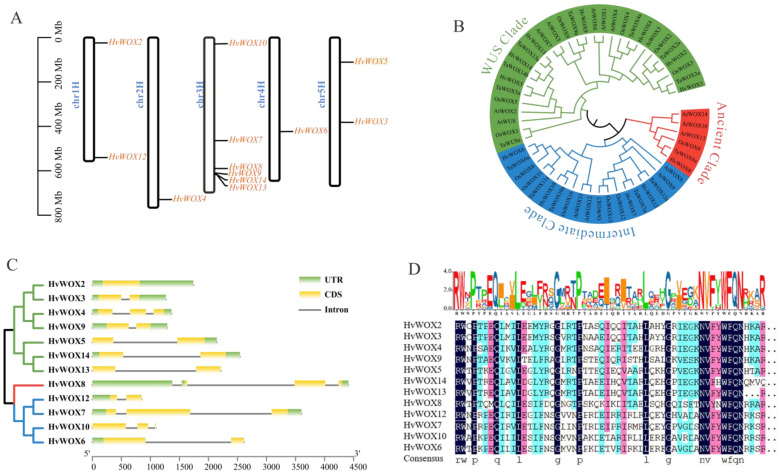
(**A**) Chromosomal distribution analysis revealed that *HvWOX* genes are unevenly distributed across five barley chromosomes, with chromosome 3 harboring the majority (6 genes). (**B**) Phylogenetic clustering of *WOX* members from barley, *Arabidopsis*, rice, and wheat. Phylogenetic analysis grouped the 12 *HvWOX* genes into three clades—WUS, ancient, and intermediate—showing evolutionary conservation with Arabidopsis, rice, and wheat *WOX* members. The green, red, and blue clusters represent the WUS clade, ancient clade, and intermediate clade, respectively. (**C**) Phylogenetic relationships and gene structures of barley *HvWOX* members. Phylogenetic tree construction based on the complete HvWOX protein sequence of barley. Exons and introns distribution is represented in barley *HvWOX* genes. Green boxes indicate UTRs, yellow boxes indicate the exons, and black lines indicate introns. (**D**) Alignment of the homeodomain sequence of barley HvWOX proteins, which confirmed the conserved helix-turn-helix-loop-helix homeodomain in all HvWOX proteins. Letters at the bottom: the amino acid that appears most frequently at that position. Black or pink background: the amino acid at that position is highly conserved across all or most sequences, meaning that the amino acid is almost identical in different HvWOX proteins. Light blue background: the amino acid at that position is conserved in some sequences but varies in others. White background: the amino acid at that position varies significantly across different sequences and has low conservation.

BLASTp searches were performed to identify *HvWOX* members in barley via the sequences of *AtWOX* members, and 12 putative *HvWOX* genes were identified in barley. The 12 *HvWOX* genes were ubiquitously present on seven chromosomes, of which chromosome 3 contained 6 genes, chromosomes 1 and 5 each contained 3 genes, chromosomes 2 and 4 contained 2 gene, and chromosomes 6 and 7 contained no genes (Figure 1A). HvWOX transporters range in length from 129 to 516 amino acids (AAs). The HvWOX protein isoelectric points ranged from 6.5 to 10.56, with two values less than 7 (Table 1). The HvWOX proteins span a range of theoretical molecular weights (MWs) from 14.74 kDa to 53.78 kDa (Table 1). The subcellular localization results revealed that nine HvWOX proteins were located in the nucleus, two proteins were located in the chloroplast, and only one protein was located in the mitochondrion (Table 1). These *HvWOX* genes were proven to contain PF00046 domains according to Pfam and SMART analyses. These results collectively highlight the structural diversity and subcellular specialization of HvWOX proteins, which may underpin their functional divergence.

### 2.2. Phylogenetic Analysis of HvWOX Genes

Having identified the chromosomal distribution and physicochemical properties of *HvWOX* genes, we next investigated their evolutionary relationships through phylogenetic analysis (Figure 1B). A phylogenetic tree was generated to explore the evolutionary relationships of *Arabidopsis*, rice, wheat, and barley *WOX* members through the use of the protein sequences of these members in MEGA 7.0. The 12 barley *HvWOXs* were classified into three clades on the basis of the relationships among the *WOX* genes in *Arabidopsis*, rice, and wheat. The ancient clade consisted of nine *WOXs*, which are orthologous to *AtWOX10*, *AtWOX13*, and *AtWOX14*, out of which only one *WOX* belongs to barley. The intermediate clade contained 19 *WOXs* and 4 *HvWOXs* orthologous to *AtWOX8*, *AtWOX9*, *AtWOX11*, and *AtWOX12*. However, the WUS clade, which was the largest clade, contained 29 *WOXs*, including 8 from *Arabidopsis* and 7 from barley (Figure 1B). These results suggested that *WOX* members in *Arabidopsis*, rice, wheat, and barley may have a close evolutionary relationship. The close phylogenetic relationship of *HvWOX* genes with *Arabidopsis*, rice, and wheat *WOX* members suggests conserved roles in plant development and stress adaptation.

### 2.3. HvWOX Gene Structures and Conserved Domains

To further explore structural diversity within the *HvWOX* family, we analyzed exon-intron architectures and conserved domains across clades (Figure 1C,D). To display the *HvWOX* gene structure in barley, we analyzed the exon—intron organization and intron phase pattern. The number of exons in the *HvWOX* genes ranged from one to three. Interestingly, *HvWOX8* has three exons, which is consistent with findings in soybean and cucumber [6,10]. The number of exons in *HvWOX* genes varies between WUS and intermediate clades. For example, *HvWOX4* contains three exons, whereas *HvWOX2* has only one exon (Figure 1C). The diverse gene structures in different phylogenetic subgroups demonstrated that genes contain different exon—intron structures to perform different functions.

WOX members containing conserved homeodomains are from different model plants [7]. Aligned sequences of the WOX proteins were used to investigate whether the central domain is conserved in HvWOX members. These HvWOX members generally contain a conserved helix-turn-helix-loop-helix homeodomain. We identified 15 highly conserved amino acid residues within the homeodomain. In the first helix, five amino acid residues, R, W, P, and Q, are highly conserved. P and L were the most conserved residues in the second helix. In the third helix, the most conserved amino acid residues were N, V, W, F, Q, and N (Figure 1D). In addition, the G amino acid residue was present in turn and in a loop. As in previous studies [10,14], each HvWOX protein contains a homeodomain, indicating that the WOX members are highly conserved. The divergence in exon–intron structures across clades, particularly between WUS and intermediate clades, implies evolutionary specialization in regulatory mechanisms.

### 2.4. Tissue Expression Patterns of HvWOXs

**Figure 2:**
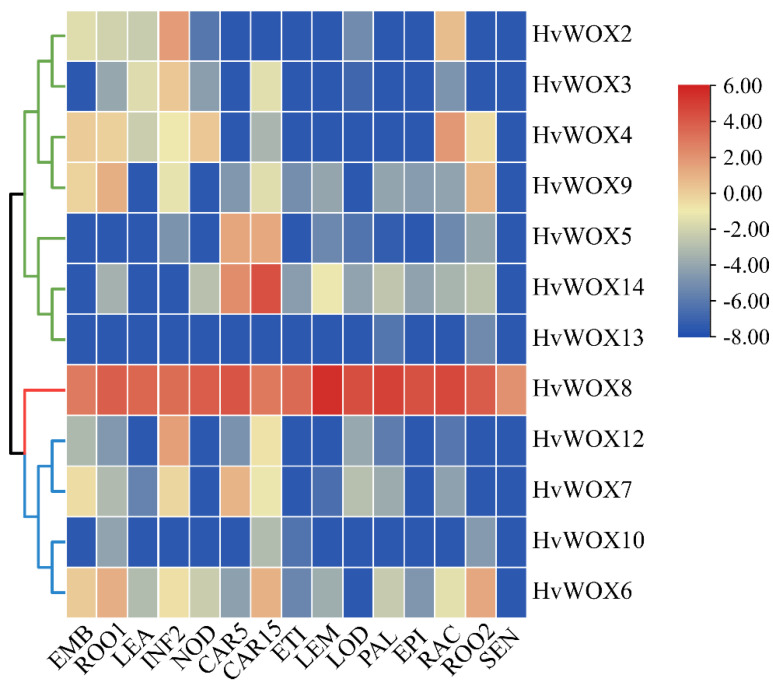
Expression profiling of *HvWOXs* in 15 tissues on the basis of transcriptomic data. Tissue-specific expression profiling demonstrated that *HvWOX8* is ubiquitously expressed, while other *HvWOX* genes show preferential expression in reproductive tissues (e.g., anthers and ovaries) and developing grains. The FPKM values were normalized by log_2_FPKM transformation. EMB, 4-day embryos; ROO1, roots from seedlings (10 cm shoot stage); LEA, shoots from seedlings (10 cm shoot stage); INF2, developing inflorescences (1–1.5 cm); NOD, developing tillers, 3rd internode (42 DAP); CAR5, developing grain (5 DAP); CAR15, developing grain (15 DAP); ETI, etiolated seedling, dark condition (10 DAP); LEM, inflorescences, lemma (42 DAP); LOD, inflorescences, lodicule (42 DAP); PAL, dissected inflorescences, palea (42 DAP); EPI, epidermal strips (28 DAP); RAC, inflorescences, rachis (35 DAP); ROO2, roots (28 DAP); SEN, sensing leaves (56 DAP).

To analyze the expression patterns of *HvWOX* genes, a tissue-specific expression profile of *HvWOXs* was constructed with the expression data of 15 barley tissues via the IPK website. As shown, *HvWOX10* and *HvWOX13* were clustered at low expression levels, whereas *HvWOX8* in the ancient clade was expressed at high levels at all stages of barley development and was not tissue specific. The rest of the family genes presented fluctuating changes in expression and were tissue specific. Among them, *HvWOX2*, *HvWOX3*, *HvWOX6*, *HvWOX7*, *HvWOX9*, and *HvWOX12* were expressed mainly in developing inflorescences, developing grains, and 4-day embryos (Figure 2). The majority of *HvWOX* genes were highly expressed in grain tissues, which suggested that these genes may be involved in plant development. The differences in the expression levels of the *WOX* genes in the tissues were similar to those reported in previous studies in soybean, wheat, and cucumber [6,10,29].

**Figure 3:**
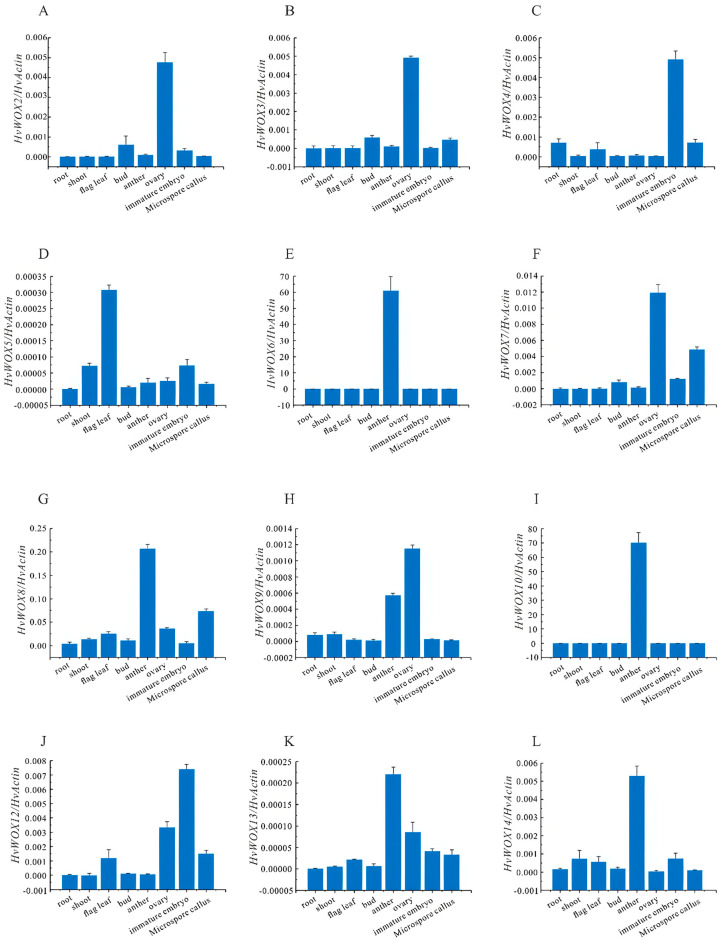
Expression patterns of *HvWOX* genes in barley. Quantitative RT–PCR validated that *HvWOX2*, *HvWOX3*, and *HvWOX7* are highly expressed in ovaries, whereas *HvWOX6* and *HvWOX13* are enriched in anthers (**A**–**L**). Tissue-specific *HvWOX* expression in barley was examined via qRT–PCR. The barley *HvActin* gene was used as an internal standard. The values are the means ± SDs of three biological replicates. Low-expression genes (Ct > 35) were excluded.

To gain further insight into the biological function of *HvWOX* genes, we analyzed their relative expression levels in the roots, shoot flag leaves, buds, anthers, ovaries, and immature embryos via quantitative RT–PCR. The results revealed that *HvWOX2*, *HvWOX3*, *HvWOX7*, *HvWOX8*, *HvWOX9*, *HvWOX12*, and *HvWOX13* were highly expressed in ovaries and that *HvWOX4*, *HvWOX5*, and *HvWOX12* were enriched in immature embryos, whereas *HvWOX6*, *HvWOX8*, *HvWOX9*, *HvWOX10*, *HvWOX13*, and *HvWOX14* were expressed mainly in anthers. Interestingly, only *HvWOX11* was highly expressed in flag leaves (Figure 3). The predominant expression of *HvWOX* genes in reproductive tissues underscores their potential roles in regulating barley embryogenesis and organogenesis.

### 2.5. Identification of Cis-Acting Elements in HvWOX Genes

**Figure 4:**
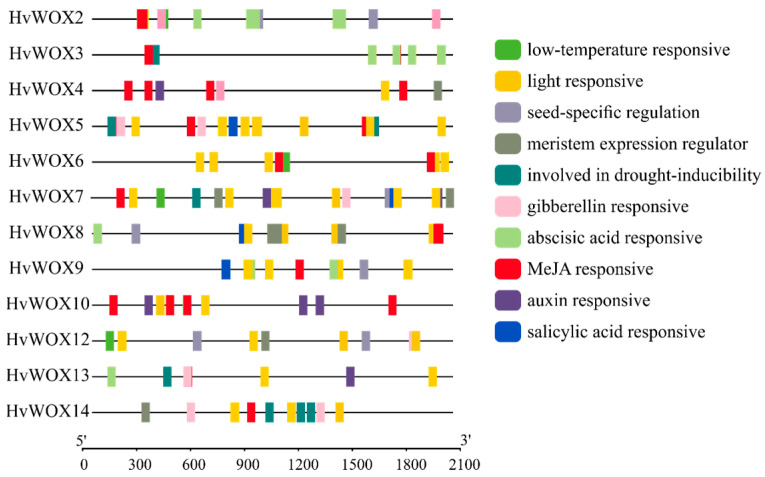
Analysis of elements in the promoters of *HvWOX* genes. Promoter analysis identified multiple stress- and hormone-responsive *cis*-elements (e.g., ABRE for ABA; AuxRE for auxin), suggesting *HvWOX* genes are regulated by abiotic stress and phytohormones. The prediction of the *cis*-elements was based on the 2 kb sequences upstream of the “ATG” of the *HvWOXs*.

Given the tissue-specific expression patterns of *HvWOX* genes, we next examined their promoter regions to identify regulatory elements that might drive these expression dynamics (Figure 4). To investigate the signal transduction of *HvWOX* genes in plants, 2 kb long upstream coding sequences of each *HvWOX* gene were extracted to analyze its *cis*-acting elements. The distribution of *cis*-elements in the *HvWOX* promoter region was analyzed, including the LTR (low-temperature response), G-box, ACE, GT1-motif, MER, C-box and Sp1 (light responsive), RY-element (seed-specific regulation), CAT-box (meristem expression regulator), MBS (drought inducibility), P-box and TATC-box (gibberellin responsive), ABRE (abscisic acid responsive), CGTCA-motif and TGACG-motif (MeJA responsive), AuxRR-core and TGA-element (auxin responsive), and TCA-element (salicylic acid responsive) (Figure 4). These results suggested that the abundance of stress- and hormone-responsive *cis*-elements in *HvWOX* promoters supports their involvement in abiotic stress signaling and phytohormone crosstalk.

### 2.6. HvWOX Gene Expression in Response to Different Hormonal Treatments in Different Tissues

Plant hormones such as auxin, cytokinin, ethylene, gibberellin (GA), and abscisic acid (ABA) have been shown to be involved in the regulation of plant development. Previous studies in rice, cucumber, and Chinese fir have shown that the expression of *WOX* is regulated by auxin and ABA [9,30,31]. To understand whether the *HvWOX* genes are regulated by plant hormones, the *HvWOX2*, *HvWOX3*, *HvWOX4*, *HvWOX7*, *HvWOX8*, *HvWOX9*, *HvWOX10*, and *HvWOX13* genes, which contain auxin or/and ABA response elements in promoters, were selected for further study. Three-day-old barley seedlings were treated with IAA and ABA, and shoots and roots were harvested at different time points. Similarly, the microspore callus was the same.

**Figure 5:**
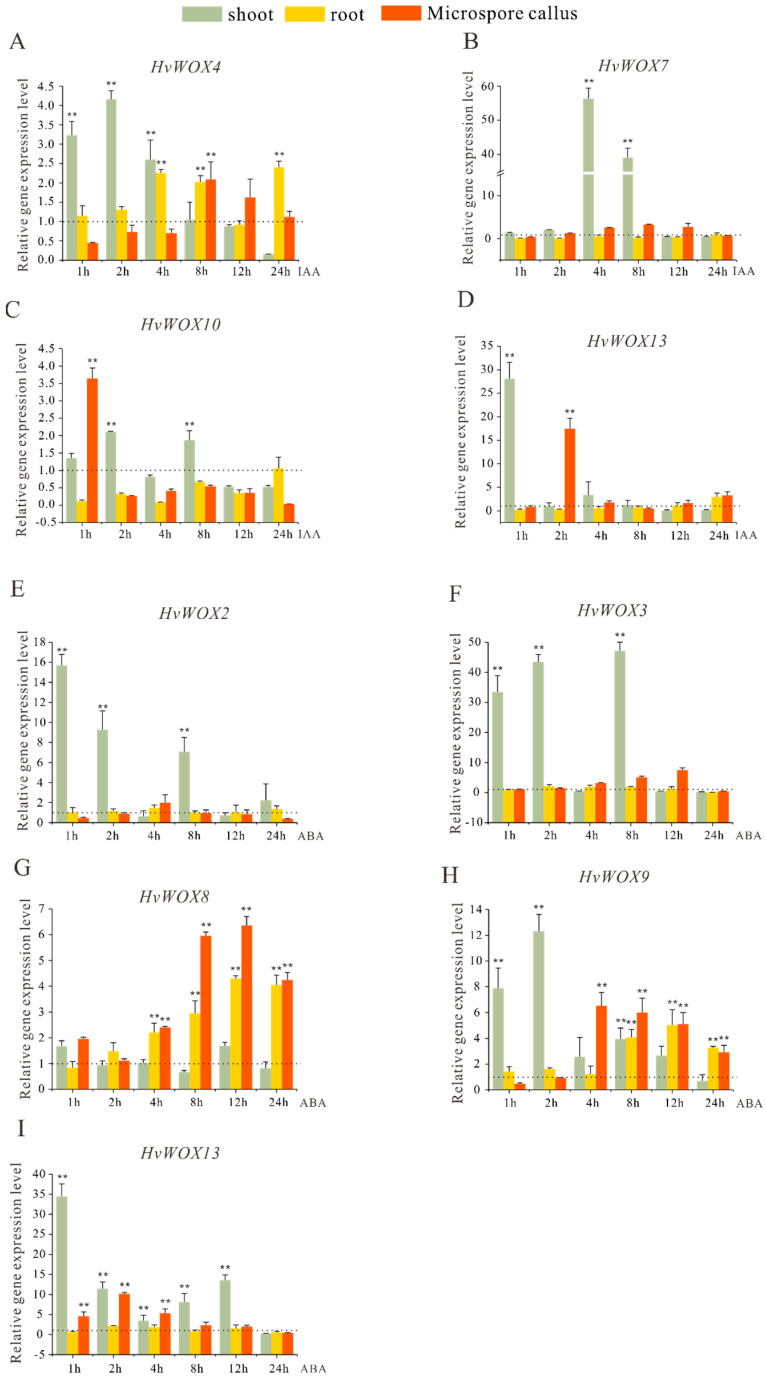
Expression profiles of four *HvWOX* genes in seedlings and microspore calli under IAA treatment. Time-course expression assays under IAA and ABA treatments revealed rapid induction of *HvWOX4* and *HvWOX13* in shoots, while *HvWOX8* exhibited delayed upregulation in roots. The expression of *HvWOX4* (**A**), *HvWOX7* (**B**), *HvWOX10* (**C**), and *HvWOX13* (**D**) was examined at 0 h, 1 h, 2 h, 4 h, 8 h, 12 h, and 24 h after 100 μM IAA treatment. The expression of *HvWOX2* (**E**), *HvWOX3* (**F**), *HvWOX8* (**G**), *HvWOX9* (**H**), and *HvWOX13* (**I**) was examined at 0 h, 1 h, 2 h, 4 h, 8 h, 12 h, and 24 h after 100 μM IAA treatment. The dotted lines indicate the expression levels of *HvWOXs* in the control. The values are the means ± SDs of three biological replicates. ANOVA was used to test significance. ** *p* < 0.01. Low-expression genes (Ct > 35) were excluded.

When treated with IAA, the expression levels of all *HvWOXs* were upregulated, but the patterns were quite different. *HvWOX4* and *HvWOX13* were rapidly induced after 1 h of IAA treatment in shoots, whereas *HvWOX7* and *HvWOX10* were induced until 2 to 4 h of IAA treatment in shoots (Figure 5A–D). In addition, only *HvWOX4* was upregulated by treatment with IAA for 4 h in the roots (Figure 5A). In addition, the expression of *HvWOX4* in microspore calli was induced by IAA treatment until 8 h, whereas *HvWOX10* and *HvWOX13* were upregulated by 1 h or 2 h of IAA treatment (Figure 5C,D).

When treated with ABA, *HvWOX2* and *HvWOX3* rapidly increased from 1 to 2 h in the shoot and even slightly changed after 4 h of ABA treatment, whereas after 8 h of ABA treatment, they were upregulated (Figure 5E,F). Moreover, *HvWOX9* and *HvWOX13* were induced by ABA, but shoot, root, and microspore calli presented different expression patterns in response to ABA (Figure 5H,I). Overall, *HvWOX8* was not sensitive to ABA in shoots, while the expression of *HvWOX8* was upregulated when roots and microspore calluses were treated with ABA for 4 h and then induced continuously throughout the experiment (Figure 5G).

Collectively, these results indicate that tissue-specific induction patterns of *HvWOX* genes by IAA and ABA suggest their functional diversification in hormone-mediated developmental processes.

**Figure 6:**
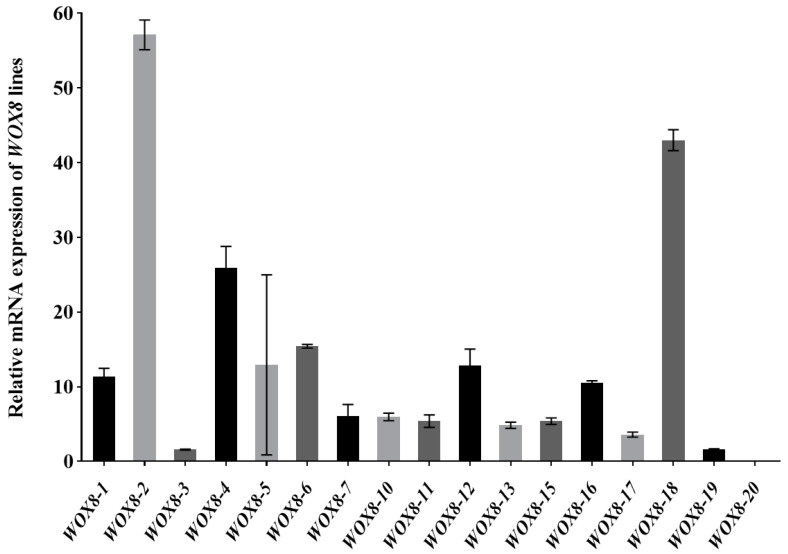
Relative mRNA expression of *HvWOX8* lines. Two independent *HvWOX8*-overexpressing *Arabidopsis* lines (WOX8-2 and WOX8-18) showed the highest transgene expression levels, confirming successful transformation. The values are presented as the means ± SDs of relative expression. The values are the means ± SDs of three biological replicates.

We conducted an assay on the expression levels of target genes in *Arabidopsis HvWOX8* gene overexpression lines. Two independent homozygous T2 lines (WOX8-2 and WOX8-18) showing the highest HvWOX8 expression levels were selected for further analysis (Figure 6).

### 2.7. HvWOX8 Improved the Tolerance of Transgenic Arabidopsis to Salt Stress by Participating in Electron Transfer During ATP Synthesis

**Figure 7:**
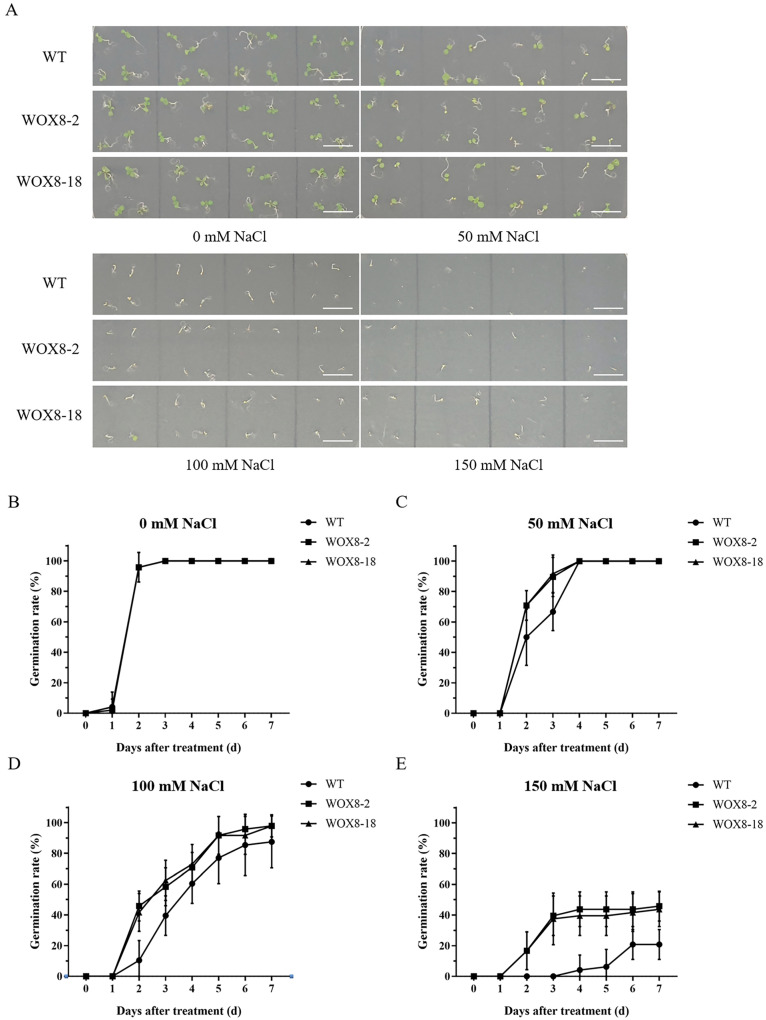
Overexpression of *HvWOX8* increased the germination rates of *Arabidopsis* seedlings under salt stress. Germination assays under salt stress (0–150 mM NaCl) demonstrated that *HvWOX8* overexpression enhances germination rates and seedling establishment compared to wild-type plants. Surface-sterilized *Arabidopsis* seeds of the wild-type and transgenic lines were sown on solid MS media supplemented with NaCl (0, 50, 100, or 150 mM). Images of their germination status were captured after 7 days (**A**), and the germination rate of the seeds was recorded every day (**B**–**E**). WT: wild-type Columbia; WOX8-2, 18: *HvWOX8 Arabidopsis* transgenic lines of the T2 generation. Bar = 1 cm. The values are the means ± SDs of three biological replicates. The overlapping lines for WT, WOX8-2, and WOX8-18 indicate no significant difference in germination rates between these genotypes.

**Figure 8:**
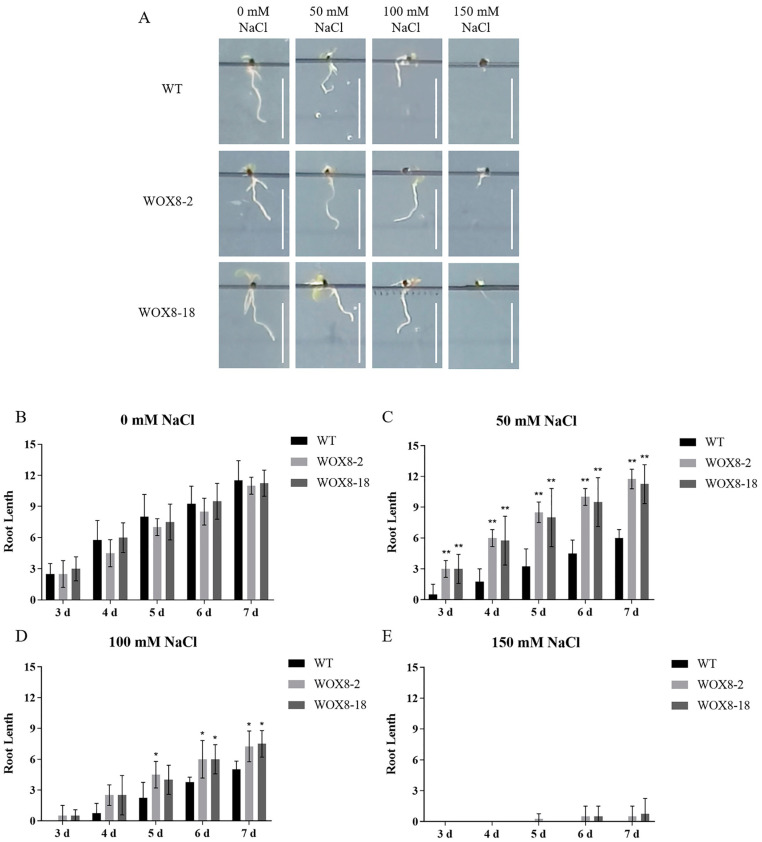
Overexpression of *HvWOX8* improved the tolerance of *Arabidopsis* seedlings to salt stress. Root elongation assays revealed that *HvWOX8*-overexpressing lines exhibit significantly longer roots under 50–100 mM NaCl, supporting its role in salt tolerance. Surface-sterilized *Arabidopsis* seeds of the wild-type and transgenic lines were sown on solid MS media supplemented with NaCl (0, 50, 100, or 150 mM), with the media placed vertically. The status of seed germination was captured after 7 days (**A**), and root length was measured from day 3 to day 7 (**B**–**E**). Four independent biological experiments were carried out to investigate the status of root growth in the WT and *HvWOX8* transgenic lines under salt stress. WT: wild-type Columbia; WOX8-2, 18: *HvWOX8 Arabidopsis* transgenic lines of the T2 generation. Bar = 1 cm. The values are the means ± SDs of three biological replicates. ANOVA was used to test significance. Asterisks indicate significant differences between the WT and *HvWOX8* transgenic lines, * *p* < 0.05; ** *p* < 0.01.

Given that *HvWOX8* is highly expressed in roots, we used the model plant *Arabidopsis thaliana* overexpressing *HvWOX8* to investigate tolerance to salt stress (Figure 7 and Figure 8).

Statistical analysis revealed that exposure to salt stress impeded the germination process of *Arabidopsis* seeds (Figure 7). Compared with the wild type, the *WOX8*-overexpressing lines presented germination patterns similar to those of the control in the absence of salt (Figure 7B). However, at a concentration of 50 mM NaCl, they germinated at a faster rate (Figure 7C), whereas at 100 mM and 150 mM NaCl, they exhibited higher germination percentages (Figure 7D,E). In terms of the effect of salt stress on the root length of *Arabidopsis*, the results indicated that there was no significant difference between the wild type and the overexpressing line under nonsalt stress conditions (Figure 8B). However, under NaCl concentrations of 50 mM (Figure 8C) and 100 mM (Figure 8D), the root length of the *HvWOX8*-overexpressing line was significantly longer than that of the wild type, demonstrating that the *HvWOX8* gene may play a role in plant salt tolerance. Nonetheless, when the NaCl concentration reached 150 mM, none of the materials grew normally (Figure 8E), indicating that the impact of the *HvWOX8* gene on salt tolerance was limited.

**Figure 9:**
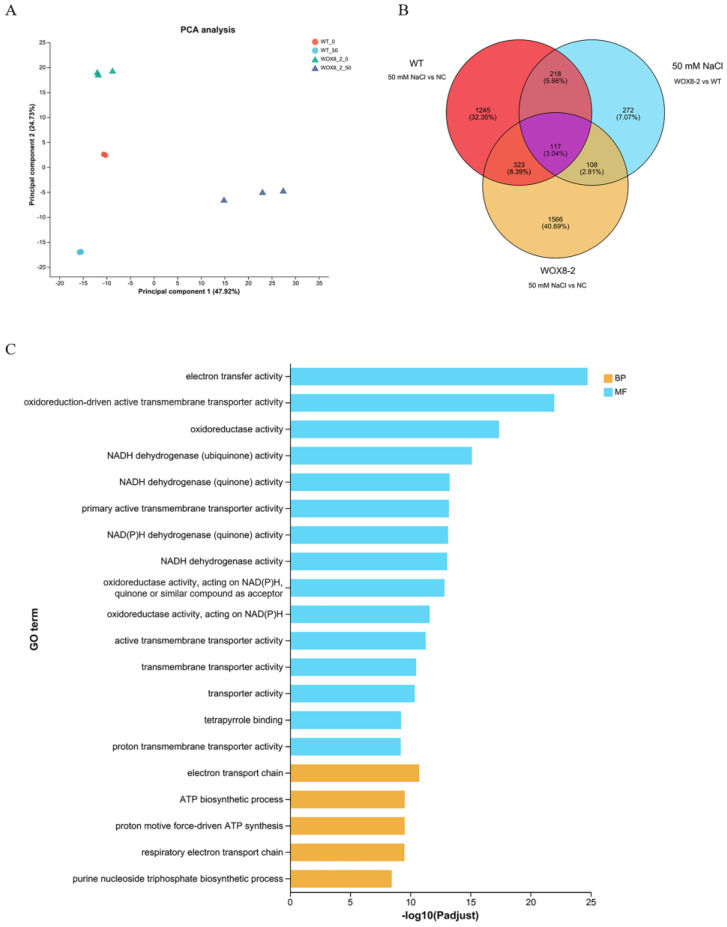
Transcriptomic analyses of WT and WOX8-2 shoots under 50 mM NaCl treatment. Transcriptomic analysis of *HvWOX8*-overexpressing lines under salt stress highlighted enriched pathways related to electron transfer, redox reactions, and ATP synthesis, providing mechanistic insights into its stress-responsive function. (**A**) Principal component analysis (PCA) of individual samples, with the numbers in parentheses indicating the percentage of total variance explained by each component. (**B**) Venn diagram showing 335 WOX8-related salt-responsive genes. (**C**) GO enrichment of the 335 WOX8-related salt-responsive genes in (**B**).

The results of the GO enrichment analysis indicated that, in the 50 mM NaCl-treated WOX8-2 group, genes or proteins related to electron transfer, redox reactions, transmembrane transport, and ATP synthesis were significantly enriched. This enrichment suggests that the 50 mM NaCl-treated WOX8-2 group may have increased activity or expression levels in these molecular functions and biological processes, highlighting a potential difference in metabolic and transport mechanisms compared with those of the 50 mM NaCl-treated WT group (Figure 9).

## 3. Discussion

### 3.1. Overview of the HvWOX Gene Family

The *WOX* gene family is among the most highly conserved gene families in plants and is pivotal for their normal development. Given that barley is the fourth largest cereal crop globally, it plays a crucial role in agricultural production and economic growth. While the WOX family has been extensively studied in various plant species, our understanding of its role in barley remains limited. Therefore, characterizing the barley *WOX* gene family holds immense potential for deepening our understanding of plant regeneration processes, particularly microspore embryogenesis.

Here, we successfully identified 12 *HvWOX* genes (Table 2) from the barley reference genome. These *HvWOX* genes were distributed on all barley chromosomes except chromosomes 6 and 7. There are additional copies of *HvWOX8* and *HvWOX10*, such as *HvWOX8.1* and *HvWOX8.2* and *HvWOX10.1* and *HvWOX10.2*, respectively [8]. Because *HvWOX8.2* was shortened compared with *HvWOX8.1* and *HvWOX10.1* and *HvWOX10.2* showed complete sequence consistency, *HvWOX8.2* and *HvWOX10.2* were removed manually. These *HvWOX* members were divided into three clades (Figure 1B, Table 1). Gene structure analysis revealed that the number of exons in *HvWOX* genes ranged from one to three, which demonstrated that these genes carry out specific functions in the barley genome (Figure 1C). Every HvWOX protein contains a conserved homeodomain, similar to the results of previous studies [9,14] (Figure 1D). Therefore, we speculated that *HvWOX* genes might play roles in activation and inhibition, similar to those in other species.

### 3.2. Putative Functions of HvWOX Genes on the Basis of Sequence Homology and Gene Expression Patterns

The *HvWOX* gene family is highly conserved in the sequence of the functional domain. Thus, it is useful to determine the function of *HvWOX* genes via analysis of tissue-specific expression. In recent years, forward and reverse genetics investigations have shown that plant *WOX* members play important roles in a variety of physiological and developmental processes. In *Arabidopsis*, the ancient clade (*AtWOX10*, *AtWOX13*, and *AtWOX14*) is expressed mainly in roots and inflorescences to regulate root development and fruit development [8]. *HvWOX8* is the only member of the ancient clade in barley. Interestingly, *HvWOX8* expression is relatively high in most tissues compared with other clade members (Figure 2 and Figure 3G). In particular, *HvWOX8* presented relatively high expression levels in anther and microspore calli (Figure 3G), suggesting that *HvWOX8* is involved in plant regeneration. However, its detailed molecular mechanism needs to be further verified. In *Arabidopsis*, the members of the intermediate clade are involved in the regulation of embryogenesis and morphological development; members of the WUS clade are involved in meristem maintenance. Here, we found that members of the intermediate clade and WUS clade presented similar expression patterns (Figure 2). The variation in exon-intron structures between clades—particularly the conserved single-exon organization in the ancient clade versus variable exon numbers in WUS and intermediate clades—suggests divergent evolutionary pressures, potentially linked to functional specialization in development or stress responses. Therefore, we speculate that these genes may share a conserved function. The quantitative qRT–PCR results further revealed that most *HvWOX* genes are expressed in ovaries, anthers, and immature embryos (Figure 3). Many genetic engineering approaches have been employed to reveal the functional importance of *WOX* genes in leaf lateral domain development [28]. In *Arabidopsis*, *AtWOX3* and *AtWOX1* are critical for leaf blade development [32]. In rice, *OsWOX3A* is a key regulator of leaf lateral development [33]. In barley, *NLD1* encodes an NS-related WOX2 protein, which we named *HvWOX3*, which plays a role in increasing organ width and in the development of marginal tissues in lateral organs [28]. Our results revealed that only *HvWOX5* was highly accumulated in flag leaves (Figure 3), which led us to investigate whether *HvWOX5* participates in leaf development. The predominant expression of *HvWOX2*, *HvWOX3*, and *HvWOX7* in ovaries and immature embryos aligns with their putative roles in reproductive development, mirroring functions of *AtWOX2* and *OsWOX11* in embryogenesis and organ regeneration. In particular, *HvWOX7*, *HvWOX8*, *HvWOX12*, and *HvWOX13* are highly expressed in microspore calli, which have strong regeneration ability, indicating that they may participate in plant regeneration. These findings provide a genetic basis for further study of barley regeneration systems. In future research, an in-depth study of the function of *HvWOX* genes will help elucidate the development pathway network related to regeneration, which is crucial in plant transformation.

### 3.3. The Response of HvWOX Genes to Auxin and ABA Provides Clues for Further Study of Their Functions

Plant hormones such as auxin, cytokinin, ethylene, gibberellin (GA), and abscisic acid (ABA) have been shown to be involved in the regulation of plant development [34]. In the past, most studies focused on the hormonal regulation of SE and plant regeneration. Analysis of the WOX promoter regions revealed that the *WOX* genes contained auxin-, GA-, and ABA-responsive elements (Figure 4). These findings suggested that *WOX* genes in barley might be involved in organ development via different plant hormones. Previous studies in rice, cucumber, and Chinese fir have shown that exogenous auxin and ABA application can modulate the expression patterns of *WOX* genes [9,30,31]. Furthermore, we detected the expression levels of *WOX* genes in three-day-old seedlings and microspore calli treated with IAA and ABA via qRT–PCR. The results revealed that auxin response elements and ABA response elements were present in *HvWOX13* and that the expression level of *HvWOX13* was greatly increased by IAA or ABA after 1 h of treatment in shoots (Figure 4 and Figure 5D,I). These findings suggested that *HvWOX13* might be involved in distinct and overlapping roles in development mediated by different plant hormones. In this study, the expression levels of most *HvWOX* genes were greatly increased by IAA or ABA after 1 h of treatment in the shoot. Nevertheless, *HvWOX* gene expression in roots was upregulated slowly under IAA or ABA treatment (Figure 5). These findings indicate that there are tissue differences in the induction of *HvWOX* genes by plant hormones. Interestingly, when microspore calluses were treated with IAA and ABA, the results revealed that the expression patterns of *HvWOXs* following IAA or ABA treatment differed (Figure 5). These findings provide a theoretical basis for our follow-up study of *HvWOX* genes cooperating with plant hormones to regulate microspore callus and plant regeneration. The expression patterns of the *HvWOX* genes in response to IAA and ABA were characterized by dual phases. Moreover, different tissues respond differently to IAA and ABA. In addition, the promoters of *HvWOX* genes also contain low-temperature-responsive elements, drought-inducible elements, and light-responsive elements, indicating that *HvWOX* genes may be involved in the response to abiotic stress. The regulatory relationship between *HvWOX* genes and abiotic stress will be further studied. These studies provide a foundation for further investigations of *HvWOX* gene functions. Further investigation of the molecular mechanisms of these genes in barley development could expand our knowledge and understanding of the roles of barley *WOX* genes.

### 3.4. HvWOX8 Enhances Plant Salt Tolerance by Responding to Hormones and Participating in Electron Transfer During ATP Synthesis

**Figure 10:**
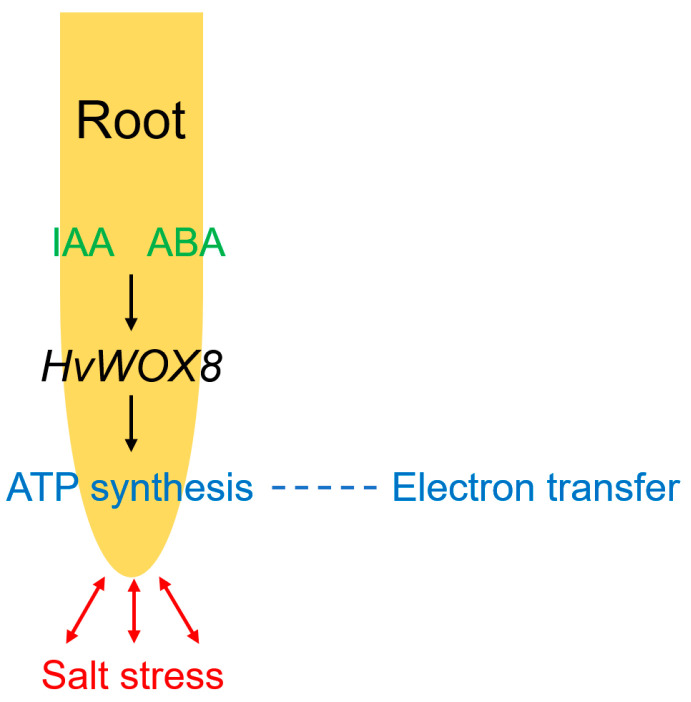
Schematic of the working hypothesis. Orange background: root. Single arrow: impact. Double arrow: salt stress to the root. Dashed line: related.

Given the high expression level of *HvWOX8* in plant roots, we aimed to investigate its potential role in root development. Considering the significant role of *WOX* genes in plant development and their involvement in hormone regulation, we hypothesize that *HvWOX8* may be related to plant stress resistance. Therefore, we used the model plant *Arabidopsis* to study the impact of *HvWOX8* on plant salt tolerance. Current research on the role of WOX genes in plant salt tolerance includes studies on *PagWOX11*/*12a* in poplar, which can directly bind to the promoter of the *PagCYP736A12* gene, regulate its expression, and effectively scavenge reactive oxygen species (ROS) to reduce hydrogen peroxide (H_2_O_2_) levels in roots under salt stress, thus increasing poplar salt tolerance [19]. A genome-wide study identified barley *WOX* genes associated with salt stress [35], further underscoring the need for functional validation. Our experiments demonstrated that *HvWOX8* can improve the salt tolerance of *Arabidopsis* seeds (Figure 7 and Figure 8), which may be related to hormone regulation (Figure 5) and electron transfer during ATP synthesis (Figure 9 and Figure 10). Additionally, transgenic barley materials are currently being transformed for further research.

## 4. Materials and Methods

### 4.1. Screening and Identification of HvWOX Family Genes in Barley

The amino acid sequences of *WOX* genes in *Arabidopsis thaliana* were downloaded from TAIR (https://www.arabidopsis.org). The identification of HvWOX proteins was carried out via a BLASTp search via the available AtWOX protein sequences against the barley genome database downloaded from the Ensembl Plants (https://sep2019-plants.ensembl.org/Hordeum_vulgare/Info/Index, accessed on 11 May 2024). The candidate sequences were submitted to the NCBI Conserved Domains (https://www.ncbi.nlm.nih.gov/Structure/bwrpsb/bwrpsb.cgi, accessed on 12 May 2024) and SMART online tools (http://smart.embl-heidelberg.de, accessed on 12 May 2024) to verify the presence of the signature homeodomain, and the sequences without the homeodomain were removed. The incomplete reading frame and redundant sequences were subsequently removed manually, after which the *WOX* genes and protein sequences in barley were finally obtained, and all manipulation of database used default parameters [36,37].

### 4.2. Analysis of the HvWOX Family and Their Chromosomal Locations

The molecular weight (MW) and theoretical isoelectric point (PI) of HvWOXs were predicted on the ExPASy website (https://web.expasy.org/compute_pi/, accessed on 5 June 2024). The predicted subcellular locations of HvWOXs are from BUSCA (http://busca.biocomp.unibo.it/, accessed on 6 June 2024). The Ensembl Plants (https://sep2019-plants.ensembl.org/Hordeum_vulgare/Info/Index, accessed on 9 June 2024) tool was used to determine the chromosomal localization of each identified gene. Conserved motifs of *HvWOXs* were analyzed and visualized via TBtools v1.095 [38].

### 4.3. Phylogenetic Analysis of HvWOX Family Genes and Sequence Alignment

The protein sequences of the OsWOXs and TaWOXs were downloaded from the RAP-DB (https://rapdb.dna.affrc.go.jp, accessed on 18 July 2024) and Ensembl (https://plants.ensembl.org/Triticum_aestivum/Info/Index, accessed on 19 July 2024) databases, respectively. MEGA 7.0 software was used to construct a phylogenetic tree of the complete protein sequence according to the methods of neighbor joining, bootstrapping, and 1000 replicates [39]. Sequence alignment of the homeodomain of HvWOX proteins was performed via DNAMAN software v7.0.2 176, and the seq-logos were visualized via TBtools v1.095 [38].

### 4.4. Data Download, Normalization, and Heatmap Generation

The raw data (FPKM) were downloaded from BARLEX (https://apex.ipk-gatersleben.de/apex/f?p=284:46:::NO:RP:P46_GENE_CHOICE:3, accessed on 6 September 2024) and normalized by log_2_FPKM transform. An expression heatmap was drawn via TBtools v1.095 [38].

### 4.5. Cis-Element Analysis of Promoter Regions of Barley WOX Genes

For *cis*-element analysis, the 2000-bp sequence from the initiation codon of *HvWOXs* was extracted. The promoter regions of *HvWOXs* were predicted and analyzed via PlantCARE (http://bioinformatics.psb.ugent.be/webtools/plantcare/html/, accessed on 12 September 2024).

### 4.6. Vector Construction and Plant Transformation

The wild-type *Arabidopsis* used for transformation was from Columbia. For the purpose of the overexpression studies, all the cloning constructs were initially amplified and inserted into the pENTR-1A vector (Invitrogen, Carlsbad, CA, USA). These vectors were subsequently recombined into a suitable destination vector via the Gateway LR reaction system (Invitrogen) [40]. These recombinant constructs were then employed to transform *Arabidopsis thaliana* of the wild-type genotype through the floral-dip method, as detailed by Clough and Bent (1998) [41]. Transgenic seedlings were initially selected on MS agar plates containing appropriate antibiotics, followed by further verification via PCR via the corresponding primers.

### 4.7. Barley Cultivation

The identification of salt-tolerant lines was conducted in field trials at the South Gate Experimental Farm of Shanghai Academy of Agricultural Sciences, covering a total area of 7.2 hm^2^, with sowing carried out in rows at a spacing of 22.5 cm and a basic seedling density of 2.25 million plants per hm^2^. The fertilization regime included base fertilizer of 150 kg/hm^2^ urea + 450 kg/hm^2^ compound fertilizer, tillering fertilizer of 112.5 kg/hm^2^ urea applied during the tillering stage, and jointing fertilizer of 112.5 kg/hm^2^ urea applied during the stem elongation stage. As for weed control, a single herbicide application was implemented throughout the entire growth period, spraying 5% Aixiu at 1200 mL/hm^2^ and 5.8% Maixi at 225 g/hm^2^.

### 4.8. HvWOX Gene Expression Analysis in Barley

The barley seeds (*Hordeum vulgare* cv. ‘Hua 30’) were sterilized with 10% commercial NaClO for 15 min and rinsed with fresh water 5 times for 5 min each. The sterilized seeds were germinated in water for 2 days and then changed to one-half Hoagland solution for another 7 days in a growth room with a photoperiod of 16/8 h, a light intensity of 200 µmol/L, a temperature of 24/18 (day/night), and a relative humidity of 60%. Total RNA was extracted via TRIzol, and cDNA for qRT-PCR amplification was synthesized from 1 μg of total RNA with PrimeScript RT Master Mix (TaKaRa, Japan). Quantitative real-time RT-PCR (qRT-PCR) was performed with an ABI PRISM 7500 Real-Time PCR System (Applied Biosystems, Waltham, MA, USA). The barley ACTIN gene was used as an internal control. Three biological replicates were analyzed for each sample. Cycle threshold (Ct) values were normalized to *HvActin*, and genes with Ct > 35 (indicating low expression) were excluded. Statistical significance was assessed via ANOVA (* *p* < 0.05; ** *p* < 0.01), and error bars represent the standard deviation (SD). The sequences of all the primers used in this study are shown in Table 2.

For gene expression pattern analysis, the barley roots, shoots, flag leaves, buds, anthers, ovaries, immature embryos, and microspore calluses were harvested with an RNA isolation kit for RNA isolation.

Three-day-old barley seedlings treated with 100 μM ABA or 100 μM IAA were used for the analysis of auxin- and ABA-induced gene expression. Seedling shoot tips and roots were separated at 0, 1, 2, 4, 8, 12, and 24 h after treatment. Moreover, microspore calli were treated with ABA or IAA in the same way.

### 4.9. Salt Tolerance Experiment of HvWOX8 in Arabidopsis

*Arabidopsis* seeds were sterilized with 5% NaClO and stored at 4 °C for three days. MS media were prepared with salt concentrations of 0, 50, 100, and 150 mM, and *Arabidopsis* seeds were spotted on the media and cultured vertically for 7 days. The length of the roots was recorded from days 3 to 7.

### 4.10. RNA Extraction and Enrichment in Arabidopsis

Total RNA was extracted via the TransZol Up reagent (TransGen Biotech, Beijing, China) according to the manufacturer’s instructions. After extraction, the RNA integrity was checked via 1.5% agarose gel electrophoresis or a fragment analyzer. The concentration and purity of the RNA were measured via a Nanodrop spectrophotometer. mRNA was enriched from the total RNA via oligo (dT) magnetic beads.

### 4.11. Transcriptome Library Construction, Quantification, and Illumina Sequencing

The transcriptome library was constructed via the VAHTS Universal V8 RNA-seq Library Prep Kit for Illumina. The constructed libraries were quantified via a Qubit 4.0 fluorometer (Thermo Fisher, Waltham, MA, USA). Sequencing was conducted on the BGISEQ MGISEQ-2000RS platform.

The RNA-seq data described in this study have been deposited in the National Center (https://www.ncbi.nlm.nih.gov/) for Biotechnology Information under BioProject PRJNA1208225 (https://www.ncbi.nlm.nih.gov/sra/PRJNA1208225, accessed on 9 January 2025).

### 4.12. Quality Control and Read Mapping

The raw paired-end reads were trimmed and quality controlled by fastp with default parameters. Then, the clean reads were separately aligned to the reference genome in orientation mode via HISAT2 software v2.2.1. The mapped reads of each sample were assembled via StringTie via a reference-based approach.

### 4.13. Differential Expression Analysis and Functional Enrichment

To identify DEGs (differentially expressed genes) between two different samples, the expression level of each transcript was calculated according to the transcripts per million reads (TPM) method. RSEM was used to quantify gene abundances. Essentially, differential expression analysis was performed via DESeq2 or DEGseq. DEGs with |log2FC| ≥ 1 and FDR < 0.05 (DESeq2) or FDR < 0.001 (DEGseq) were considered significantly differentially expressed genes. In addition, functional enrichment analysis, including GO and KEGG analyses, was performed to identify which DEGs were significantly enriched in GO terms and metabolic pathways at a Bonferroni-corrected *p* value < 0.05 compared with the whole-transcriptome background. GO functional enrichment and KEGG pathway analyses were carried out via Goatools v1.4.4 and Python SciPy software v3.9.14, respectively.

## Figures and Tables

**Table 1:** Complex characteristics of *HvWOX* genes and proteins with various features.

Gene Name	Gene ID	Genomic Location	Clade	Protein Length (AA)	Molecular Weights (kDa)	Isoelectric Point	Subcellular Localization
*HvWOX2*	HORVU1Hr1G010580	24,444,001–24,445,742	WUS Clade	211	22.88	9.40	nucleus
*HvWOX3*	HORVU5Hr1G049190	381,765,625–381,766,908	WUS Clade	186	18.83	7.85	nucleus
*HvWOX4*	HORVU2Hr1G113820	729,806,496–729,808,073	WUS Clade	234	25.30	9.60	nucleus
*HvWOX5*	HORVU5Hr1G022120	111,001,243–111,003,388	WUS Clade	276	29.56	9.80	nucleus
*HvWOX6*	HORVU4Hr1G051530	423,508,136–423,511,456	Intermediate Clade	314	32.42	6.52	mitochondrion
*HvWOX7*	HORVU3Hr1G060950	464,417,446–464,421,050	Intermediate Clade	516	53.78	8.07	nucleus
*HvWOX8*	HORVU3Hr1G080660	589,829,423–589,834,968	Ancient Clade	208	23.42	7.11	nucleus
*HvWOX9*	HORVU3Hr1G085050	610,834,437–610,835,788	WUS Clade	209	24.25	7.06	nucleus
*HvWOX10*	HORVU3Hr1G013290	28,673,837–28,674,948	Intermediate Clade	261	28.21	6.50	chloroplast
*HvWOX12*	HORVU1Hr1G087950	540,697,582–540,698,431	Intermediate Clade	129	14.74	10.56	chloroplast
*HvWOX13*	HORVU3Hr1G086450	617,085,484–617,087,698	WUS Clade	274	30.20	9.80	nucleus
*HvWOX14*	HORVU3Hr1G086430	616,993,938–616,996,482	WUS Clade	283	31.61	9.54	nucleus

**Table 2:** Primers used in this study.

Gene Name	Direction	Sequence
*HvActin*	Forward	CGACAATGGAACCGGAATG
	Reverse	CCCTTGGCGCATCATCTC
*HvWOX2*	Forward	CGCAGATCCAGCAGATCACG
	Reverse	GAGAGGAGGTGGTGGCTCAT
*HvWOX3*	Forward	GCGGCTCCTCCTCCTACTAC
	Reverse	CATGAAGCAGCTGGTAGCGT
*HvWOX4*	Forward	GGATCGAGGGCAAGAACGTC
	Reverse	GAGGAAGAGTCGAGGGTGCT
*HvWOX5*	Forward	ATGCAACGGACAAGCGATGT
	Reverse	GCTCCCTGGTAGTACACGGT
*HvWOX6*	Forward	GTTCCAGAACCGTCGTTCCC
	Reverse	CGGATGGAAGACCGATGGTG
*HvWOX7*	Forward	GGATCCAGCAAACCGCTCAA
	Reverse	GGCGACGAACTCGTCCAAAT
*HvWOX8*	Forward	GGAGGCCACAAGATCACAGC
	Reverse	GGTCTCCGAGATCTGTCCGT
*HvWOX9*	Forward	GCAGATCCAGCGGATTTCCA
	Reverse	CGGTCTCTTCCTCGTTGCTG
*HvWOX10*	Forward	CGTGACGCCAACGTGTTCTA
	Reverse	CCGTACTGCATGGCGTTGTA
*HvWOX12*	Forward	CGTCCTCCAACAAGCACTGG
	Reverse	TTGCTCATGTCAGGCTGGTG
*HvWOX13*	Forward	GTTACATGACAGGGCCAGCA
	Reverse	CCATGCGCCATCGTCTCTAC
*HvWOX14*	Forward	GCATGCAACGGAGAAGCAAC
	Reverse	ATCGTCTCCGCACTTGGGTA

## Data Availability

The RNA-seq data described in this study have been deposited in the National Center for Biotechnology Information (https://www.ncbi.nlm.nih.gov/) under BioProject PRJNA1208225 (https://www.ncbi.nlm.nih.gov/sra/PRJNA1208225).
